# Frailty or sarcopenia: which is a better indicator of mortality risk in older adults?

**DOI:** 10.1136/jech-2024-222678

**Published:** 2024-10-11

**Authors:** Aline Fernanda de Souza, Paula Camila Ramírez, Dayane Capra de Oliveira, Roberta de Oliveira Máximo, Mariane Marques Luiz, Maicon Luis Bicigo Delinocente, Maria Claudia Bernardes Spexoto, Andrew Steptoe, Cesar De Oliveira, Tiago da Silva Alexandre

**Affiliations:** 1 Postgraduate Program in Physical Therapy, Federal University of São Carlos, São Carlos, Brazil; 2 Escuela de Fisioterapia, Universidad Industrial de Santander, Bucaramanga, Colombia; 3 Postgraduate Program in Gerontology, Federal University of São Carlos, São Carlos, Brazil; 4 Food, Nutrition and Health Postgraduate Program, Federal University of Grande Dourados, Dourados, Brazil; 5 Department of Epidemiology and Public Health, University College London, London, UK; 6 Gerontology Department, Federal University of São Carlos, São Carlos, Brazil

**Keywords:** MORTALITY, AGING, EPIDEMIOLOGY, GERONTOLOGY

## Abstract

**Background:**

Despite the different conditions, frailty and sarcopenia overlap regarding their common link: the assessment of walking speed and muscle strength. This study aimed to compare the frailty phenotype to the sarcopenia using different cut-off points for low grip strength to determine which better identifies mortality risk over a 14-year follow-up period.

**Methods:**

4597 participants in the English Longitudinal Study of Ageing. Frailty was measured using the Fried phenotype. Sarcopenia (European Working Group on Sarcopenia in Older People 2) was defined using different cut-off points for low grip strength (<36, <32, <30, <27 and <26 kg for men and <23, <21, <20 and <16 kg for women), low skeletal muscle mass index (<9.36 kg/m² for men and<6.73 kg/m² for women) and slowness (gait speed: ≤0.8 m/s). Cox models were run and adjusted for sociodemographic, behavioural and clinical factors.

**Results:**

When the coexistence of frailty and sarcopenia is considered, only the cut-off points <36 kg for men and <23 kg for women to define low grip strength identified the risk of mortality among individuals classified as having probable sarcopenia (HR=1.17, 95% CI 1.02 to 1.34), sarcopenia (HR=1.31, 95% CI 1.07 to 1.60) and severe sarcopenia (HR=1.62, 95% CI 1.33 to 1.96). In this situation, frailty identified the mortality risk (HR=1.49, 95% CI 1.22 to 1.81), whereas pre-frailty did not. Sarcopenia using other cut-off points for defining low grip strength did not identify mortality risk.

**Conclusion:**

Sarcopenia using <36 kg for men and <23 kg for women as cut-off points seems to be better than the frailty phenotype for identifying the risk of mortality in older adults.

**WHAT IS ALREADY KNOWN ON THIS TOPIC**

- Although sarcopenia and frailty often coexist, they are conceptually different. While both share the same central core of musculoskeletal decline, the conditions have distinct terminologies for their components and different cut-off points to define them in terms of slower walking speed and poorer muscular strength. Currently, some studies point to the association between frailty and mortality and between sarcopenia and mortality. However, no studies have compared the two conditions, individually or collectively, to determine the real power of effect of each condition and its association with mortality. Thus, based on the similarity of mortality risk, it is necessary to compare frailty and sarcopenia to assist health professionals with screening the elderly population in different clinical contexts.

**WHAT THIS STUDY ADDS**

- When the coexistence of frailty and sarcopenia is considered, sarcopenia with cut-off points <36/23 kg is a better indicator of mortality risk.

**HOW THIS STUDY MIGHT AFFECT RESEARCH, PRACTICE OR POLICY**

- In addition to being a better indicator of mortality, the use of higher cut-off points to define low strength in sarcopenia seems to be better, as it enables the early identification of sarcopenia and greater time for the implementation of treatment strategies that could result in the greater success of interventions.

## Introduction

 Sarcopenia and frailty have been linked to a range of adverse outcomes in older adults, including death.^1 2^ Between updating the *European Working Group on Sarcopenia in Older People* (EWGSOP) 1 and 2 and the present day, research groups have sought a way of defining and diagnosing sarcopenia.^3 4^ However, none have had the same success in disseminating the concept as *EWGSOP1* and, subsequently, *EWGSOP2*. Given the large quantity of evidence on the determinant role of low muscle strength^4^ in the occurrence of adverse outcomes in older people, the operational definition of sarcopenia proposed by the *EWGSOP2* began to consider the diagnosis of the condition in the occurrence of the combination of low muscle strength and low muscle mass, with low physical performance considered an aggravating factor.^4^

Although the operational definition of sarcopenia by the *EWGSOP2* is widely used in research and clinical practice, there needs to be a consensus on the best cut-off point for determining low muscle strength.^2^ At least five different cut-off points are found to define low strength in men and women, respectively: <26 and <16 kg; <27 and <16 kg; <30 and <20 kg; <32 and <21 kg; and <36 and <23 kg, which hinders the establishment and comparison of the prevalence of the condition in different locations as well as the establishment of associations with the main adverse outcomes in older people.^4^,^8^

Regarding frailty, the phenotype and Frailty Index (FI) assess this condition differently. While the FI is composed of a long list of clinical conditions and diseases that require a medical assessment,^9^ the frailty phenotye^10^ consists of the assessment of five components: slowness, weakness, unintentional weight loss, exhaustion and low physical activity level. Comparing the two frailty assessment models, the complexity of information and the high number of items in the FI could hinder its use, as many items are more easily assessed in a hospital setting.^9^ The assessment based on the phenotype is considered a more practical approach in the clinic. It is used in studies that assess community-dwelling older people, as the components can be investigated in the primary care setting.

Because the frailty phenotype requires assessing five components to define the syndrome, the assessment of sarcopenia seems more advantageous, requiring the evaluation of only three items. Moreover, muscle mass estimated using a validated equation has been considered reliable and safe,^11 12^ offering greater practicality and agility in day-to-day clinical practice. Furthermore, sarcopenia and frailty have something in common: the decline of skeletal muscle function. Thus, both share the components of slow walking speed (slowness) and low strength (weakness).^13^

However, while slowness is defined in the *EWGSOP2* consensus as walking speed ≤0.8 m/s and low strength is defined as grip strength <27 kg for men and <16 kg for women,^4^ slowness and weakness are described in the frailty phenotype by the sample distribution (20% slowest and weakest), with cut-off points for defining slowness stratified by sex and average height and weakness stratified by sex and quartiles of the body mass index (BMI).^10^

Few studies have compared frailty and sarcopenia as predictors of the risk of death. For example, a recent English study assessed 101 983 individuals between 37 and 73 years of age to investigate associations between mortality and sarcopenia, frailty, cachexia and malnutrition in 10 years. The highest risk of death was found among frail individuals who had two or more of the other conditions investigated (396%), followed by frail sarcopenic individuals (27%) and individuals with frailty alone (13%).^14^ However, only 8% were non-frail in the study, and sarcopenia was not investigated in an isolated manner, impeding the determination of which would be the better predictor of death. Furthermore, another study analysed frailty and sarcopenia separately as risk factors for mortality in 208 Belgian men between 70 and 85 years of age, followed up for 15 years. Although the difference was small, frail individuals had a greater risk of mortality compared with sarcopenic individuals (164% vs 150%).^1^ Despite the similar risk and the fact that frailty has indicators of the decline of skeletal muscle function, the assessment of the two conditions is performed little, so it is necessary to identify which should be the priority in clinical practice.

Therefore, the present study aimed to compare frailty to sarcopenia according to the *EWGSOP2* consensus using different cut-off points to define low strength and determine which would better identify the mortality risk in a 14-year follow-up period.

## Methods

The data for the present study were from the *English Longitudinal Study of Ageing* (*ELSA*). Details on the *ELSA* can be found in another publication.^15 16^

This study used data from wave 2 of the *ELSA* study (2004) as the baseline, which was the first time anthropometric measures and physical performance were collected. Among the 6182 participants ≥60 years of age, 1585 were excluded due to missing information for the definition of sarcopenia, frailty or the covariables, resulting in a final analytical sample of 4597 participants.

### Muscle strength assessment

Grip strength was measured using a handgrip dynamometer.^17^,^19^ Low strength was considered using different cut-off points: <36, <32, <30, <27 and<26 kg for men and<23, <21, <20 and<16 kg for women.^4^,^8^ Detailed information can be found in the online supplemental material (Muscle strength assessment section).

### Skeletal muscle mass assessment

Skeletal muscle mass was estimated using Lee’s equation.^11 12^ The skeletal muscle mass index values used to define low muscle mass were <9.36 kg/m^2^ for men and<6.73 kg/m^2^ for women.^8 19 20^ Detailed information can be found in the online supplemental material (Skeletal muscle mass assessment section).

### Physical performance assessment

Walking speed assessed physical performance and was considered low when ≤0.8 m/s.^421^,^23^ Detailed information can be found in the online supplemental material (Physical performance assessment section).

### Sarcopenia

Sarcopenia was defined based on the *EWGSOP2* using different cut-off points for grip strength.^4^ The online supplemental material (Sarcopenia section) provides detailed information.

### Frailty

The adapted Fried phenotype defined frailty.^1017 24^,^28^ The online supplemental material (Frailty section) provides detailed information.

All measures used in the definition and diagnosis of sarcopenia and frailty were assessed at baseline.

### Mortality

Mortality data were obtained from the English mortality system. All deaths in the baseline sample were recorded during the 14-year follow-up period.

### Covariates

Variables described in the literature as associated with mortality were considered, such as age, sex, race, marital status, total family wealth, schooling,^29 30^ smoking, alcohol intake, physical activity level,^17 27 28^ self-reported medical diagnosis of systemic arterial hypertension, diabetes mellitus, cancer, lung disease, heart disease, stroke, falls, depressive symptoms,^26 31^ memory score^32^ and BMI.^24^ Detailed information can be found in the online supplemental material (Covariates section).

### Statistical analyses

The characteristics of the sample at baseline were expressed as means, SDs and proportions. We investigated all deaths in the sample occurring in the 14-year follow-up period. Follow-up time for the deceased was calculated by the difference between the date of death (day/month/year) and the initial interview date. For those who lived until the end of the follow-up period, time was calculated by the difference between the last recorded data (interview) and data from the initial interview.

Using different cut-off points for grip strength, the Kaplan-Meier estimator was used to analyse survival curves and explore associations between mortality, frailty and sarcopenia. Differences between curves were analysed using the log-rank test. Cox regression models were run to investigate associations between sarcopenia and mortality and frailty and mortality. Moreover, to identify whether sarcopenia or frailty was the better predictor of death, Cox models were run with the two conditions included in the same model and estimating HRs and respective 95% CIs. The models were controlled by sociodemographic, behavioural, clinical and anthropometric variables.

All models were compared using the concordance (C) index. A C index of 0.5 indicates a low-performance model, whereas a value of 1 indicates a model with perfect prediction.^33 34^ The Stata 17.0 statistical package was used for all analyses, with a p value <0.05 indicative of statistical significance.

## Results

A total of 2669 participants died during the 14-year follow-up period. The sample was composed predominantly of women (55.2%), white individuals (99.9%) with a conjugal life (65.4%) and a low level of schooling (57.1%). In terms of behavioural characteristics, the most significant portions of the sample were former smokers (51.3%) with frequent alcohol intake (40.8%) and were physically active (95.4%). Hypertension was the most prevalent among the health conditions investigated (47.5%), followed by heart disease (25.2%). The most considerable portion of the sample was overweight (44.4%) (table 1).

**Table 1:** Sociodemographic characteristics, behavioural characteristics, health conditions and anthropometry at baseline for 4597 participants in the ELSA study (2004)

Sociodemographic characteristics	ELSAn=4597
Age (mean), SD	70.6 (7.4)
Sex (female), %	55.2
Race (white), %	99.8
Marital status (without conjugal life), %	34.6
Total family wealth (quintiles), %	
Fifth quintile (highest)	21.8
Fourth quintile	21.4
Third quintile	20.6
Second quintile	19.6
First quintile (lowest)	16.5
Not declared	0.1
Schooling, %	
>13 years	22.0
12–13 years	20.9
0–11 years	57.1
**Behavioural characteristics**	
Smoking, %	
Non-smoker	36.7
Former smoker	51.3
Smoker	12.0
Alcohol intake, %	
Non-drinker or intake up to once per week	18.8
Intake two to six times per week	40.8
Daily intake	30.8
Not declared	9.6
Physical activity (inactive), %	4.6
**Health conditions**	
Systemic arterial hypertension (yes), %	47.5
Diabetes mellitus (yes), %	8.9
Cancer (yes), %	9.2
Lung disease (yes), %	18.5
Heart disease (yes), %	25.2
Stroke (yes), %	5.3
Falls (yes), %	30.6
Depressive symptoms (yes), %	13.5
Memory score (mean), SD	9.5 (3.4)
**Anthropometry**	
Body mass index, %	
Eutrophic (≥18.5 kg/m² BMI<25 kg/m²)	27.2
Underweight (<18.5 kg/m²)	0.8
Overweight (≥25 kg/m² BMI<30 kg/m²)	44.4
Obesity (≥30 kg/m²)	27.6

Note: Data isare expressed in proportions, as well as means and standard deviationsSDs.

BMIbody mass indexELSAEnglish Longitudinal Study of Ageing

Irrespective of the cut-off points used to define low strength, most of the sample did not have sarcopenia. However, when the cut-off point of <36/23 kg was used to determine low strength, the prevalence of probable sarcopenia (28.2%), sarcopenia (6.6%) and severe sarcopenia (7.8%) increased, as expected. Regarding frailty, 42.9% of the sample were considered pre-frail and 10.9% were deemed frail (table 2).

**Table 2:** Prevalence of frailty and sarcopenia according to different grip strength cut-off points for defining low strength at baseline of 4597 participants of the ELSA study (2004)

Construct of sarcopenia	ELSAn=4597
Construct <26/16 kg, %	
No sarcopenia	90.5
Probable sarcopenia	5.6
Sarcopenia	1.1
Severe sarcopenia	2.8
Construct <27/16 kg, %	
No sarcopenia	89.8
Probable sarcopenia	6.2
Sarcopenia	1.2
Severe sarcopenia	2.8
Construct <30/20 kg, %	
No sarcopenia	78.8
Probable sarcopenia	13.3
Sarcopenia	2.7
Severe sarcopenia	5.2
Construct <32/21 kg, %	
No sarcopenia	71.8
Probable sarcopenia	17.8
Sarcopenia	4.0
Severe sarcopenia	6.4
Construct <36/23 kg, %	
No sarcopenia	57.4
Probable sarcopenia	28.2
Sarcopenia	6.6
Severe sarcopenia	7.8
**Frailty**	
Non-frail, %	46.2
Pre-frail, %	42.9
Frail, %	10.9

Note: Data expressed in proportions.

ELSAEnglish Longitudinal Study of Ageing

Severe sarcopenia was only associated with a greater risk of mortality when the cut-off points for defining low strength were <30/20 kg (HR=1.30, 95% CI 1.09 to 1.56), <32/21 kg (HR=1.42, 95% CI 1.19 to 1.69) and<36/23 kg (HR=1.83, 95% CI 1.53 to 2.19) for men and women, respectively. Probable sarcopenia (HR=1.28, 95% CI 1.14 to 1.45) and sarcopenia (HR=1.35, 95% CI 1.11 to 1.65) were only associated with a greater risk of mortality when the cut-off point for defining low strength was <36/23 kg for men and women, respectively. Moreover, both pre-frailty and frailty were associated with a greater risk of mortality (HR=1.23, 95% CI 1.09 to 1.38 and HR=1.78, 95% CI 1.49 to 2.11, respectively). Therefore, for the analyses in which sarcopenia and frailty were assessed in separate models, severe sarcopenia defined by grip strength <36/23 kg identified a greater risk of death (table 3).

**Table 3:** Cox regression models for associations between different grip strength cut-off points for defining low strength in constructs of sarcopenia and mortality as well as between frailty and mortality during a 14-year follow-up of 4597 older adults from the ELSA study

	Frailty	Construct of sarcopenia <26/16 kg	Construct of sarcopenia <27/16 kg	Construct of sarcopenia <30/20 kg	Construct of sarcopenia <32/21 kg	Construct of sarcopenia <36/23 kg
	Adjusted HR (95% CI)	Adjusted HR (95% CI)	Adjusted HR (95% CI)	Adjusted HR (95% CI)	Adjusted HR (95% CI)	Adjusted HR (95% CI)
Sarcopenia						
No sarcopenia		1.00	1.00	1.00	1.00	1.00
Probable sarcopenia		1.04 (0.86 to 1.26)	1.02 (0.85 to 1.22)	1.05 (0.91 to 1.21)	1.04 (0.92 to 1.19)	1.28 (1.14 to 1.45)**
Sarcopenia		0.94 (0.65 to 1.36)	0.96 (0.67 to 1.37)	0.92 (0.70 to 1.20)	1.08 (0.86 to 1.35)	1.35 (1.11 to 1.65)*
Severe sarcopenia		1.19 (0.95 to 1.48)	1.21 (0.97 to 1.49)	1.30 (1.09 to 1.56)*	1.42 (1.19 to 1.69)*	1.83 (1.53 to 2.19)**
Frailty						
Non-frail						
Pre-frail	1.23 (1.09 to 1.38)**					
Frail	1.78 (1.49 to 2.11)**					
C index	0.7781	0.7758	0.7758	0.7762	0.7766	0.7784

Note: HR: hazard ratio; : confidence interval; Models adjusted by age, sex, race, marital status, schooling, total family wealth, smoking, alcohol intake, physical activity level, systemic arterial hypertension, diabetes mellitus, cancer, lung disease, heart disease, stroke, falls, depressive symptoms, memory score and body mass index (BMI).*p; **p.

*P<0.05; **p<0.001.

ELSAEnglish Longitudinal Study of Ageing

**Figure 1:**
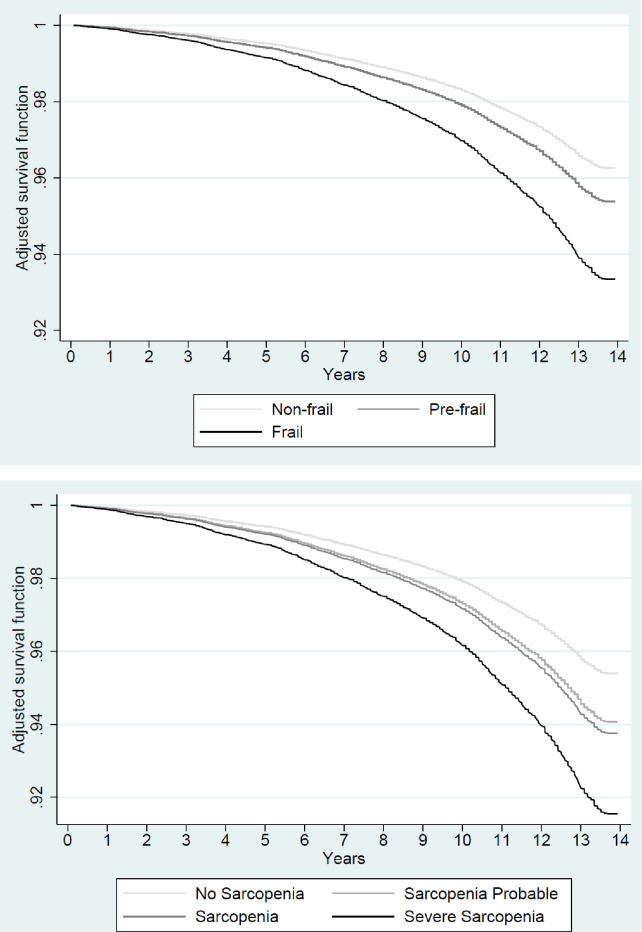
Survival analysis of frailty and sarcopenia (with low strength defined with grip strength <36 kg for men and <23 kg for women) based on the final Cox proportional hazards model, calculated for the reference/baseline values of the covariates in the study. The baseline values were as follows: ages 60–69 years, women, white race, with conjugal life, schooling >13 years, in the highest quintile of total family wealth, non-smoker, non-drinker or intake up to once per week, physically active lifestyle, no systemic arterial hypertension, no diabetes mellitus, no cancer, no heart disease, no lung disease, no stroke, no falls, no depressive symptoms, highest memory score and normal weight.

When frailty and sarcopenia were included in the same model, pre-frailty was only associated with a greater risk of death when sarcopenia was defined by handgrip cut-off points <26/16 kg (HR=1.24, 95% CI 1.10 to 1.40), <27/16 kg (HR=1.24, 95% CI 1.10 to 1.40), <30/20 kg (HR=1.27, 95% CI 1.12 to 1.44) and <32/21 kg (HR=1.26, 95% CI 1.11 to 1.44) to define low strength. Frailty was associated with a greater risk of death irrespective of the cut-off points adopted to define low strength. However, the model with the handgrip cut-off point <36/23 kg to define low strength was the only one in which pre-frailty was not associated with mortality. Furthermore, in this model, the participants with probable sarcopenia (HR=1.17, 95% CI 1.02 to 1.34), sarcopenia (HR=1.31, 95% CI 1.07 to 1.60) and severe sarcopenia (HR=1.62, 95% CI 1.33 to 1.96) were at risk of death irrespective of frailty. Lastly, this model demonstrated that individuals with severe sarcopenia were at greater risk of death compared with those with frailty (HR=1.49, 95% CI 1.22 to 1.81) (table 4) (figure 1).

**Table 4:** Comparative analyses of different constructs of sarcopenia and frailty in the same model as a risk factor for mortality in a 14-year follow-up of 4597 participants of the ELSA study

	Construct of sarcopenia <26/16 kg	Construct of sarcopenia <27/16 kg	Construct of sarcopenia <30/20 kg	Construct of sarcopenia <32/21 kg	Construct of sarcopenia <36/23 kg
	Adjusted HR (95% CI)	Adjusted HR (95% CI)	Adjusted HR (95% CI)	Adjusted HR (95% CI)	Adjusted HR (95% CI)
Sarcopenia					
No sarcopenia	1.00	1.00	1.00	1.00	1.00
Probable sarcopenia	0.91 (0.75 to 1.10)	0.88 (0.74 to 1.06)	0.88 (0.75 to 1.02)	0.87 (0.75 to 1.01)	1.17 (1.02 to 1.34)*
Sarcopenia	0.89 (0.61 to 1.28)	0.90 (0.63 to 1.28)	0.81 (0.62 to 1.07)	0.95 (0.75 to 1.20)	1.31 (1.07 to 1.60)*
Severe sarcopenia	1.00 (0.79 to 1.25)	1.01 (0.81 to 1.26)	1.05 (0.87 to 1.28)	1.13 (0.93 to 1.37)	1.62 (1.33 to 1.96)**
Frailty					
Non-frail	1.00	1.00	1.00	1.00	1.00
Pre-frail	1.24 (1.10 to 1.40)**	1.24 (1.10 to 1.40)**	1.27 (1.12 to 1.44)**	1.26 (1.11 to 1.44)**	1.12 (0.98 to 1.27)
Frail	1.81 (1.51 to 2.17)**	1.82 (1.51 to 2.19)**	1.85 (1.52 to 2.25)**	1.82 (1.49 to 2.23)**	1.49 (1.22 to 1.81)**
C index	0.7782	0.7783	0.7785	0.7787	0.7793

Note: HR: hazard ratio; : confidence interval; Models adjusted by age, sex, race, marital status, schooling, total family wealth, smoking, alcohol intake, physical activity level, systemic arterial hypertension, diabetes mellitus, cancer, lung disease, heart disease, stroke, falls, depressive symptoms, memory score and body mass index (BMI). *p; **p.

*P<0.05; **p<0.001.

ELSAEnglish Longitudinal Study of Ageing

In the analysis comparing those individuals included in the study to those excluded at baseline due to missing information, the excluded individuals were older, mainly were married, non-white, had lower wealth, had lower schooling, were more inactive and consumed more alcohol. Systemic arterial hypertension, diabetes mellitus, heart disease, stroke, falls, depressive symptoms and a poor memory score were more prevalent among the excluded individuals (online supplemental table 1).

## Discussion

The construct of the *EWGSOP2* using grip strength cut-off points <36 kg for men and <23 kg for women to define low strength was better than the frailty phenotype for identifying the risk of mortality in older people.

The study by De Buyser *et al*^1^ was the only investigation to assess sarcopenia and frailty separately as risk factors for mortality, following 208 Belgian men 70–85 years of age for 15 years. Sarcopenia was assessed using the Foundation Criteria of the National Institutes of Health, and frailty was assessed using the FI of the Study of Osteoporotic Fractures. Despite the small difference, frail individuals were at greater risk of mortality than sarcopenic individuals (164% vs 150%).^1^ However, the study was restricted to the male sex and the sample comprised only 208 individuals. Moreover, no analysis incorporating both constructs in a single model was conducted, which would have demonstrated which association was more robust. This impedes the comparison of results to those of the present investigation.

Using another analytical approach, Landi and collaborators followed up 364 frail participants between 80 and 85 years of age to assess sarcopenia, defined based on the *EWGSOP1*, as a risk factor for death in frail individuals. The authors found that frail individuals with sarcopenia had a greater risk of all-cause mortality (132%) compared with those who were only frail.^35^ With this approach, however, it is impossible to examine each condition’s individual risk. In our study, when sarcopenia and frailty were analysed in the same model, sarcopenia was defined with higher cut-off points of grip strength to classify low strength as a better predictor of death compared with frailty, highlighting its predictive capacity.

The existence or absence of compromised physical functioning (represented by low muscle strength and slowness in both sarcopenia and the frailty phenotype) is a central component in determining vulnerability and the risk of adverse outcomes in older people.^36^,^38^ However, it should be stressed that low strength is always a component of the diagnosis of probable sarcopenia, and slowness always characterises the severity of the disease.^4^ In contrast, low strength and slowness may or may not be present in pre-frailty or frailty as components of the phenotype.^10^ Thus, although an individual may be pre-frail or frail without the components of slowness and low muscle strength,^10^ the literature has demonstrated that these two indicators, which are present in the assessment of sarcopenia, are more strongly linked to death,^36^,^40^ which would explain our results.

On the other hand, although unintentional weight loss is an essential component of the frailty phenotype^10^ regarding the risk of mortality, one should remember that Lee’s equation includes weight for estimating muscle mass.^11^ Therefore, an individual diagnosed with sarcopenia^4^ has an essential indicator of low muscle mass. In contrast, those considered pre-frail or frail may or may not have the unintentional weight loss component of the phenotype.^10^ In clinical practice, the assessment of sarcopenia, even when using Lee’s equation, would be more agile and complete for identifying the risk of mortality than the assessment of the frailty phenotype.

Regarding comparing grip strength cut-off points for defining low strength in the consensus of sarcopenia and the association with mortality in older adults, our results agree with the findings described by Spexoto and collaborators.^8^ The authors analysed a cohort of 6182 English people 60 years of age or older who were followed up for 14 years and found that the cut-off points of <36 kg for men and <23 kg for women for the definition of low strength were the best predictor of the risk of mortality.^8^ The use of higher cut-off points for defining low strength in sarcopenia seems to be better in long follow-up periods, as it enables the early identification of sarcopenia, allowing more time for the implementation of treatment strategies, which can result in the greater success of the intervention. Lower cut-off points were identified in other studies with shorter follow-up periods, which limits the window of opportunity for interventions that may be more effective.^6^

This study has potential limitations and strong points that should be acknowledged. Our findings must be considered in the context of community-dwelling individuals aged 60 years or older. Caution should be exercised when interpreting the results in the clinical/hospital setting and long-term care facilities/nursing homes. Another limitation could be the determination of skeletal muscle mass using an equation. However, this does not compromise our findings, as the equation has been validated, achieved a good coefficient of determination when using one of the gold standard methods (magnetic resonance) and is a more practical way to estimate muscle mass in clinical contexts with scarce resources. The exclusion of individuals with missing data at baseline may have been a source of bias, as these individuals had worse socioeconomic, behavioural and clinical conditions associated with mortality. It is challenging to infer whether the exclusion of these individuals led to an overestimation or underestimation of our associations, as the measures of frailty and sarcopenia could not be used with these individuals, despite knowing that the more prevalent conditions in the individuals excluded are associated with both frailty and sarcopenia. This study also has strong points, such as using standardised tools for identifying frailty syndrome, including a large representative sample of community-dwelling English older adults and a long 14-year follow-up period. Moreover, this is the first study to compare frailty and sarcopenia defined by the *EWGSOP2* using different grip strength cut-off points for defining low strength recommended in the literature in models adjusted by a broad gamut of covariables associated with mortality.

## Conclusion

The *EWGSOP2* construct using grip strength cut-off points of <36 kg for men and <23 kg for women to define low strength was better than the frailty phenotype for identifying mortality risk in older people. Assessing sarcopenia using the *EWGSOP2* construct with grip strength <36/23 kg to define low strength proved more complete and could offer greater agility in clinical practice for identifying mortality risk.

## supplementary material

10.1136/jech-2024-222678 online supplemental file 1

## Data Availability

All data relevant to the study are included in the article or uploaded as supplementary information.
