# Supplementary material for: Frailty or sarcopenia: which is a better indicator of mortality risk in older adults?
Source: J Epidemiol Community Health. 2024 Oct 11;79(2):e222678. doi: 10.1136/jech-2024-222678 (PMC11730048; doi:10.1136/jech-2024-222678)
Supplement: online supplemental file 1 [file jech-79-2-s001.pdf]

Frailty phenotype or sarcopenia according to the *EWGSOP2*: which is a better indicator of mortality risk in older adults?

Supplementary material

1. Supplementary Methods

Muscle strength assessment section

Muscle strength was estimated based on grip strength measured using a handgrip dynamometer (Smedley; range: 0 to 100 kg). The test was performed with the participant standing, arms alongside the body and elbow flexed at 90 degrees.[1] Three trials were performed with the dominant hand, respecting a one-minute rest between trials. The highest value was considered for the analyses.[2,3] To determine whether different grip strength cut-off points used in the definition of low strength would alter the associations between sarcopenia and mortality, low strength was defined when grip strength was <36, <32, <30, <27 and <26 kg for men and <23, <21, <20 and <16 kg for women.[4-8]

Skeletal muscle mass assessment section

Skeletal muscle mass (SMM) was determined using Lee's equation.[9] Al-Gindan and collaborators (2014) validated this equation using whole-body magnetic resonance. They found an adjusted coefficient of determination of 0.85 for men and women.[10] Spexoto and collaborators (2022) also used this equation to estimate SMM in a study investigating the association between mortality and different cut-off points for sarcopenia over 14 years.[8] After the estimate of SMM, the skeletal muscle mass index (SMMI) (kg/m^2) was calculated. The cut-

off points for defining low SMMI were based on the 20th percentile of the sample distribution. Thus, low muscle mass was considered when the SMMI was <9.36 kg/m^2 for men and <6.73 kg/m^2 for women.[11-12]

Physical performance assessment section

Walking speed was used for the assessment of physical performance. The participants were instructed to walk 2.4 meters on a flat surface without assistance. The total distance in meters was divided by time in seconds for the conversion into meters/second (m/s).[13-15] Two trials were performed, and the best time was considered for analysis. Low physical performance was defined as ≤ 0.8 m/s.[6,16]

Sarcopenia section

Sarcopenia was defined based on the *EWGSOP2* using different cut-off points for grip strength. The participants were classified into four groups: no sarcopenia, probable sarcopenia (only low strength), sarcopenia (low strength + low muscle mass) or severe sarcopenia (low strength + low muscle mass + low physical performance).[6]

Frailty section

Frailty was defined by the adapted Fried phenotype (2001).[17] Unintentional weight loss was defined based on the body mass index (BMI) < 18.5 kg/m^2.[18,19] Exhaustion was defined by an affirmative answer to one of the following statements: 'I feel that everything I did was an effort in the last week'; 'I could not get "going" in the last week'. These statements were taken from the Center for Epidemiologic Studies Depression Scale (CES-D).[20] Weakness was defined by the lowest quintile of grip strength stratified by sex in each BMI quartile. The lowest quintile of walking speed is defined as slowness based on the average of two measures (2.4 m) stratified

by height (average) and sex.[17] Low physical activity level was determined using three questions taken from the Physical Activity and Sedentary Behaviour Assessment Questionnaire (PASBAQ) used in the Health Survey for England (HSE) and based on the frequency and intensity with which the participants practised vigorous, moderate, and light physical activity (more than once per month, once per week, one to three times per week or never). Those who reported never performing moderate-intensity physical activity were considered to have a low physical activity level.[21,22] Participants with three or more of the components described above were considered frail, those with one or two were considered pre-frail, and those with no components were considered non-frail.[17]

Covariates section

Variables described in the literature as associated with mortality were considered control variables and measured at the baseline of the present study (2004). The sociodemographic variables were age (continuous), sex, race (white or non-white), marital status (with or without a conjugal life), total family wealth (quintiles) and schooling following the English standard (0–11; 12–13; > 13 years).[23,24]

The behavioural characteristics of interest were smoking (non-smoker, former-smoker, or smoker), alcohol intake (non-drinker or intake up to once per week, intake two to six times per week or daily intake).[23] For physical activity, the individuals were classified as inactive (vigorous or moderate, once per week, one to three times per month, hardly ever or never; any mild physical activity) or active (vigorous or moderate, more than once per week) based on the Physical Activity and Sedentary Behaviour Assessment Questionnaire validated by the Health Survey for England.[21,22]

Health conditions were assessed based on self-reports of systemic arterial hypertension, diabetes mellitus, cancer, lung disease, heart disease, stroke, and the occurrence of falls in the previous year. Depressive symptoms were defined by a score of ≥ 4 points on the *Center for Epidemiological Studies-Depression Scale*.[25] Memory was assessed using the word list test, divided into two parts: immediate recall (the participant hears 10 words and repeats them) and delayed recall (the participant is asked to recall the words after three minutes). The score is obtained by the number of correctly remembered words in both parts of the test and ranges from 0 to 20, with higher scores indicating a better memory performance.[26] For body mass index, individuals were classified as eutrophic (≥ 18.5 BMI < 25 kg/m²), underweight (BMI < 1.5 kg/m²), overweight (≥ 25 BMI < 30 kg/m²) or with obesity (BMI ≥ 30 kg/m²).[18]

2. <u>Supplementary Table</u>

Supplementary Table 1. Sociodemographic, behavioural, health conditions and anthropometry characteristics among individuals included and excluded in the sample due to missing data at baseline, the ELSA study (2004).

**Sociodemographic characteristics**	***ELSA* n = 4,597**	***ELSA* n = 1,585**
Age (mean), (SD)	70.6 (7.4)	73.6 (8.7)*
Sex (female), %	55.2	56.7
Race (white), %	99.8	96.0*
Marital status (without conjugal life), %	34.6	43.4*
Total family wealth (quintiles), %		
Fifth quintile (highest)	21.8	28.9
Fourth quintile	21.4	21.9
Third quintile	20.6	18.8*
Second quintile	19.6	14.9*
First quintile (lowest)	16.5	15.4*
Not declared	0.1	0.1
Schooling, %		
>13 years	22.0	16.4
12 – 13 years	20.9	14.7
0 – 11 years	57.1	68.9*
**Behavioral characteristics**		
Smoking, %		
Non-smoker	36.7	33.8
Former smoker	51.3	50.9
Smoker	12.0	15.3*
Alcohol intake, %		
Non-drinker or intake up to once per week	18.8	21.4
Intake two to six times per week	40.8	26.4*
Daily intake	30.8	22.3*
Not declared	9.6	29.9*
Physical activity (inactive), %	4.6	19.8*
**Health conditions**		
Systemic arterial hypertension, (yes) %	47.5	52.2*
Diabetes mellitus (yes), %	8.9	13.8*
Cancer (yes), %	9.2	9.2
Lung disease (yes), %	18.5	19.7
Heart disease (yes), %	25.2	32.1*
Stroke (yes), %	5.3	11.6*
Falls (yes), %	30.6	38.2*
Depressive symptoms (yes), %	13.5	21.4*
Memory score, (mean) (SD)	9.5 (3.4)	8.1 (3.8)*
**Anthropometry**		
Body mass index, %		
Eutrophic ($\geq$ 18.5 kg/m² BMI < 25 kg/m²)	27.2	27.9
Underweight (<18.5 kg/m²)	0.8	0.5
Overweight ($\geq$ 25 kg/m² BMI < 30 kg/m²)	44.4	36.9
Obesity ($\geq$ 30 kg/m²)	27.6	34.7

Note: Data is expressed in proportions, as well as means and standard deviations.

3. Supplementary References

1. Banks J, Breeze E, Lessof CNJ. Retirement, health and relationships of the older population in England: the 2004 English Longitudinal Study of Ageing. London: Institute for Fiscal Studies; 2006.
2. Al Snih S, Markides KS, Ottenbacher KJ et al. Hand grip strength and incident ADL disability in elderly Mexican Americans over a seven-year period. Aging Clin Exp Res 2004;16(6):481–486.
3. Bohannon RW, Magasi S. Identification of dynapenia in older adults through the use of scores. Muscle & nerve 2015;51(1):102–105.
4. Cruz-Jentoft AJ, Bahat G, Bauer J et al. Sarcopenia: revised European consensus on definition and diagnosis. Age Ageing 2019;48:16-31.
5. Alley DE, Shardell MD, Peters KW et al. Grip strength cutpoints for the identification of clinically relevant weakness. J Gerontol A Biol Sci Med Sci 2014;69:559–66.
6. Lauretani F, Russo CR, Bandinelli S et al. Age-associated changes in skeletal muscles and their effect on mobility: an operational diagnosis of sarcopenia. J Appl Physiol 2003;95:1851–60.
7. Delinocente MLB, de Carvalho DHT, Maximo RO et al. Accuracy of different handgrip values to identify mobility limitation in older adults. Arch Gerontol Geriatr 2021;94:104347.
8. Spexoto MCB, Ramirez PC, Maximo RO et al. European Working Group on Sarcopenia in Older People 2010 (EWGSOP1) and 2019 (EWGSOP2) criteria or slowness: which is the best predictor of mortality risk in older adults? Age and Ageing 2022;51:1–10.
9. Lee RC, Wang Z, Heo M et al. Total-body skeletal muscle mass: development and cross-validation of anthropometric prediction models. Am J Clin Nutr 2000;72(3):796–803.
10. Al-Gindan YY, Hankey C, Govan L et al. Derivation and validation of simple equations to predict total muscle mass from simple anthropometric and demographic data. Am J Clin Nutr 2014;100(4):1041–51.
11. Delmonico MJ, Harris TB, Lee JS et al. Alternative definitions of sarcopenia, lower extremity performance, and functional impairment with aging in older men and women. J Am Geriatr Soc, 55: 769–74, 2007.
12. Coin A, Sarti S, Ruggiero E et al. Prevalence of sarcopenia based on different diagnostic criteria using DEXA and appendicular skeletal muscle mass reference values in an Italian population aged 20 to 80. J Am Med Dir Assoc, 14: 507–12, 2013.
13. Guralnik JM, Ferrucci L, Simonsick EM, Salive ME, Wallace RB. Lower-extremity function in persons over the age of 70 years as a predictor of subsequent disability. N Engl J Med 1995; 332: 556–62.
14. Guralnik JM, Simonsick EM, Ferrucci L, Glynn RJ, Berkman LF, Blazer DG, et al. A short physical performance battery assessing lower extremity function: association with self-reported disability and prediction of mortality and nursing home admission. Journal of gerontology. 1994 Mar;49(2):M85-94.
15. Guralnik JM, Ferrucci L, Pieper CF, Leveille SG, Markides KS, Ostir G V, et al. Lower extremity function and subsequent disability: consistency across studies, predictive models, and value of gait speed alone compared with the short physical performance battery. The journals of gerontology Series A, Biological sciences and medical sciences. 2000 Apr;55(4):M221-31.
16. Cruz-Jentoft AJ, Baeyens JP, Bauer JM, Boirie Y, Cederholm T, Landi F, et al. Sarcopenia: European consensus on definition and diagnosis. Age and Ageing. 2010;39(4):412–23.
17. Fried, LP Tangen CM, Walston J et al. Frailty in Older Adults: Evidence for a Phenotype. Journal of Gerontology: MEDICAL SCIENCES, 56(3): 146–156, 2001.

18. Organization WH. Obesity: Preventing and Managing the Global Epidemic. World Health Organization; 2000.
19. Oliveira DC, Máximo RO, Ramírez PC et al. Is slowness a better discriminator of disability than frailty in older adults? Journal of Cachexia, Sarcopenia and Muscle, 12: 2069–2078, 2021.
20. Radloff LS. The CES-D scale: a self-report depression scale for research in the general population. Appl Psychol Measur, 1:385–401, 1977.
21. Craig R, Mindell J, Hirani V, Joint Health Surveys Unit (Great Britain), Great Britain, National Health Service, et al. Health survey for England 2008: physical activity and fitness. London: National Centre for Social Research with permission of The NHS Information Centre; 2009.
22. Scholes S, Coombs N, Pedisic Z et al. Age- and sex-specific criterion validity of the health survey for England Physical Activity and Sedentary Behavior Assessment Questionnaire as compared with accelerometry. Am J Epidemiol 2014;179(12):1493–502.
23. Alexandre T da S, Scholes S, Ferreira Santos JL, Duarte YA de O, de Oliveira C. The combination of dynapenia and abdominal obesity as a risk factor for worse trajectories of IADL disability among older adults. Clinical nutrition (Edinburgh, Scotland). 2018 Dec;37(6 Pt A):2045–53.
24. Banks J, Kumari M, Smith JP, Zaninotto P. What explains the American disadvantage in health compared with the English? The case of diabetes. Journal of epidemiology and community health, 66(3):259–64, 2012.
25. Gallagher D, Kiss A, Lanctot K, Herrmann N. Depressive symptoms and cognitive decline: a longitudinal analysis of potentially modifiable risk factors in community dwelling older adults. J Affect Disord., 190:235–240, 2016.
26. Huppert FA et.al. Cognitive function. In Banks J, Breeze E, Lessof C, Nazroo J, eds. Retirement, Health and Relationships of the Older Population in England: The 2004 English Longitudinal Study of Ageing, 217–242, 2006.
